# Interplay between heart rate and its variability: a prognostic game

**DOI:** 10.3389/fphys.2014.00347

**Published:** 2014-09-12

**Authors:** Jerzy Sacha

**Affiliations:** Department of Cardiology, Regional Medical CenterOpole, Poland

**Keywords:** heart rate, heart rate variability, heart rate dynamics, prediction, correlation, risk factor

In the last decades, heart rate (HR) and heart rate variability (HRV) were extensively investigated in various clinical and laboratory settings and proved to be significant risk factors for different outcomes and patients' populations (Kannel et al., 1987; Bravi et al., 2011; Antoni et al., 2012). During that time, a number of new methods permitting to explore different aspects of HR variability and dynamics have been implemented (Task Force of the European Society of Cardiology, and the North American Society of Pacing and Electrophysiology, 1996; Schmidt et al., 1999; Bauer et al., 2006). However, most of them yield parameters which are essentially associated with HR (Tsuji et al., 1996; Cygankiewicz et al., 2004; Sacha and Pluta, 2005, 2008; Lewek et al., 2009; Sacha, 2013), therefore, they actually carry information not only on the variability but also on HR itself (Sacha, 2014a,b). In fact it is hard to ascertain which of these two (i.e., HR or variability) really matters in the prognostic significance of HRV (Sacha, 2013, 2014a,b). Recently, a new approach which enables to weaken (or even eliminate) or strengthen the association between HR and its variability has been proposed (Sacha et al., 2013a). The principles of this method are simple, i.e., by division of RR interval tachograms by the corresponding average RR intervals, the variability of RR intervals of slow HR is attenuated, but that of fast HR is relatively amplified and the resulting HRV loses its correlation with HR. On the other hand, by multiplication of RR interval tachograms by their average RR intervals, the variability of slow HR is boosted but that of fast HR is relatively suppressed, and consequently, the correlation between HRV and HR is growing. By division or multiplication by higher powers of average RR intervals stronger effect on the HRV/HR relationship can be achieved (Sacha et al., 2013a). Moreover, by division by very high powers, this relationship may even be inverted, i.e., from negative to positive one (Figure 1A) (Sacha et al., 2013b).

**Figure 1:**
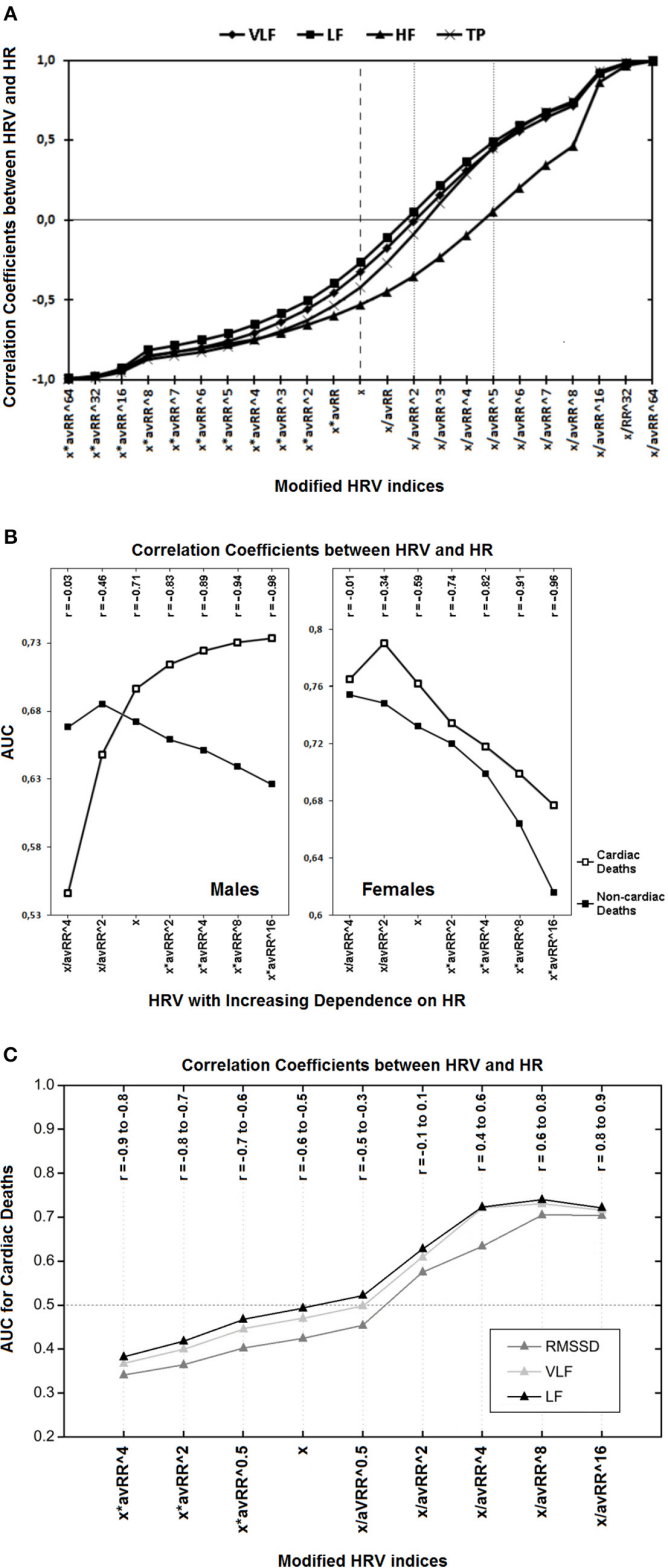
**(A)** Correlation coefficients between different modified spectral HRV indices and HR are presented. The “x” denotes standard HRV indices which all are inversely associated with HR; multiplication by different powers of the corresponding average RR interval (avRR) increases this negative relationship (the left half of the diagram); but division by avRR to different powers diminishes the HRV dependence on HR and may even inverse this relationship, i.e., from negative to positive one (the right half of the diagram). Of note, to become HR-independent (i.e., to achieve insignificant correlation coefficients) VLF, LF and TP have to be divided by avRR^∧^2, but HF by avRR^∧^5 (see respective markers on dotted lines)—this is due to the fact that HF is initially more dependent on HR (compare respective markers on the dashed line). HF, high frequency component; LF, low frequency component; VLF, very low frequency component; TP, total power (Reprinted from Sacha et al., 2013b). **(B)** The prediction performance (i.e., AUC, area under receiver-operator characteristic curves) for different classes of modified HRV indices (i.e., very low frequency components of HRV spectrum) and their correlation coefficients (r) with HR in males and females are depicted. The “x” denotes standard HRV indices, other indices were calculated by division or multiplication of “x” by different powers of the corresponding average RR interval (avRR). In the first two classes (i.e., x/avRR^∧^4 and x/avR^∧^2), the HRV dependence on HR was weakened, while it was strengthened in the last four classes (i.e., x^*^avRR^∧^2, x^*^avRR^∧^4, x^*^avRR^∧^8 and x^*^avRR^∧^16). As HRV is becoming more dependent on HR (i.e., from the first to the seventh class), its predictive ability increases in men for cardiac death but decreases for non-cardiac one, while in women, it decreases for both outcomes. It is noteworthy that HR was a strong predictor of cardiac death and a weak predictor of non-cardiac death in males, however, in females HR did not predict any mode of death (Reprinted with modification from Sacha et al., 2014). **(C)** Predictive performance for cardiac mortality (i.e., AUC, area under receiver-operator characteristics curves) for different classes of modified HRV indices and their correlation coefficients (r) with HR, during the recovery after exercise test are presented. The “x” denotes standard HRV indices, other indices were calculated by multiplication or division of “x” by different powers of the corresponding average RR interval (avRR). The AUC < 0.5 indicates that higher HRV is related with worse prognosis but AUC > 0.5 means that higher HRV is associated with better prognosis. Standard HRV indices (i.e., x) are negatively correlated with HR and after multiplication by different powers of avRR this negative correlation becomes tighter, along with the improvement in their predictive ability (i.e., AUC is getting lower and lower)—of note, higher values of these indices are related with worse prognosis. However, the division by different powers of avRR makes HRV indices either independent on HR (i.e., x/avRR^∧^2) or positively correlated with HR (i.e., x/avRR^∧^4, x/avRR^∧^8 and x/avRR^∧^16) along with the increase in their predictive power—higher values of these indices are associated with better prognosis (i.e., AUC > 0.5). LF, low frequency component; RMSSD, root mean square successive differences; VLF, very low frequency component (Reprinted with modification and permission from Pradhapan et al., 2014).

Recent studies with implementation of this method have shown that HR may have different impact on the prognostic ability of HRV for different outcomes (Sacha et al., 2013c). In general, it seems that for populations and events where HR is a significant risk factor the enhancement of its impact improves the prognostic value of HRV, however, for groups and outcomes where HR is not or is a weak risk factor, the exclusion of its influence increases the HRV prediction capacity (Sacha et al., 2013c, 2014; Sacha, 2014a,b). In particular, such phenomena were observed in the study addressing HRV and HR in different genders after myocardial infarction (Sacha et al., 2014). In other words, HR was a strong risk factor of cardiac death in men and strengthening its influence on HRV boosted the HRV prediction performance for cardiac mortality, conversely, HR was a poor predictor of non-cardiac death in male subgroup and weakening its impact augmented the HRV prognostic value for non-cardiac mortality. However, HR did not predict any outcomes in females and the exclusion of its influence improved the HRV prognostic ability for every mode of death in women (Figure 1B) (Sacha et al., 2014).

The concept of such prognostic HRV and HR interaction has been recently confirmed in a large group of patients undergoing exercise tests (i.e., 1288 participants) (Pradhapan et al., 2014). The study showed that HR right before exercise was not a risk factor of death and elimination of its influence improved the predictive capability of the respective HRV, conversely, HR during recovery phase was a significant mortality predictor and the enhancement of its impact augmented the respective HRV prognostic performance (Pradhapan et al., 2014).

Low HRV and high HR are usually associated with worse prognosis, however, there are also situations where such a coincidence represent favorable prospect—this can be seen during exercises (Dewey et al., 2007). In the aforementioned study by Pradhapan et al., higher HRV and lower HR (i.e., below 125 bpm) during recovery phase were related with an increased risk of both cardiac and non-cardiac death (Pradhapan et al., 2014). However, if one inverts the HRV/HR relationship from negative to positive one (i.e., by division of HRV indices by high powers of their corresponding average RR intervals), higher HRV represents good prognosis—Figure 1C (Pradhapan et al., 2014).

Thus, the interplay between HRV and HR turns out to be quite complicated. However, the unraveling of this remarkable game, by using the method of strengthening or weakening the HRV/HR dependence (Sacha et al., 2013a), may yield valuable prognostic information (Sacha et al., 2013c, 2014; Pradhapan et al., 2014; Sacha, 2014a,b). This is particularly important in women, in whom HR is a weak (or even is not) risk factor and actually can cover the prognostic value of HRV—yet, this detrimental effect may be eliminated by the removal of HRV dependence on HR (Sacha et al., 2014).

Currently, it is hard to conclude how to practically employ the aforementioned method in clinical settings. However, a concept of a separate approach to HR and its variability should enable us to determine which of the two quantities presents higher predictive performance for a given population and outcome. Probably, it will give us possibilities to increase the prognostic value of HRV by the suitable modification of its relationship with HR. It should be stressed that such a method may be employed to any other HR dynamics analysis which parameters are significantly correlated with HR (Sacha, 2014a,b). The first experiences with using this approach are encouraging, however, the concept requires further investigations in various clinical situations.

## Conflict of interest statement

The author declares that the research was conducted in the absence of any commercial or financial relationships that could be construed as a potential conflict of interest.
